# How does pregnancy affects drug disposition of lithium? A retrospective observational cohort study

**DOI:** 10.1192/j.eurpsy.2025.320

**Published:** 2025-08-26

**Authors:** M. L. Imaz, M. Torra, K. Langohr, E. Poch, D. Soy, L. Garcia-Esteve, E. Vieta, R. Martín-Santos

**Affiliations:** 1 Unit of Perinatal Mental Health, Department of Psychiatry and Psychology, Hospital Clinic; 2 Institut d’Investigacions Biomèdiques August Pi i Sunyer (IDIBAPS); 3 Department of Medicine, University of Barcelona; 4 Pharmacology and Toxicology Laboratory, Biochemistry and Molecular Genetics Service, Biomedical Diagnostic Center (CBD), Hospital Clinic; 5 University of Barcelona; 6 Department of Statistics and Operational Research, Universitat Politècnica de Catalunya; 7 Nephrology and Renal Trasplant Service; 8 Division of Medicines, Pharmacy Service; 9 Psychiatry and Psychology, Hospital Clinic; 10 Centro de Investigación Biomédica en Red en SAlud Mental (CIBERSAM), Barcelona, Spain

## Abstract

**Introduction:**

Lithium is used as a first-line treatment in perinatal bipolar disorder. Lithium is almost exclusively renal eliminated without undergoing metabolization. Renal changes associated with pregnancy are responsible for alterations in lithium pharmacokinetics that may impact lithium efficacy and toxicity in mother. Characterization of the trajectory of lithium disposition during the perinatal period (previous year of pregnancy, pregnancy and first year postpartum) is necessary to monitoring and dose adjustments to prevent bipolar symptom recurrence while minimizing adverse effects.

**Objectives:**

To characterize the disposition of lithium during the perinatal period ant to evaluate whether changes in serum lithium concentration are consistent with changes in renal function (creatinine).

**Methods:**

Women treated with lithium carbonate and referred to the perinatal psychiatry out-patient clinic of a single tertiary university hospital (November 2006 -December 2018), were evaluated for eligibility to participate in this retrospective observational cohort study (HCB/2020/1305). The basis of this study was all samples analysed in the same laboratory for lithium during perinatal period obtained at steady-state and predose. Serum creatinine concentrations measurements were also extracted. Lithium concentrations were determined by means of an AVL 9180 electrolyte analyzer based on the ion- selective electrode (ISE) measurement principle. Detection limit (LoD) was 0.1 mEq/L and limit of quantification (LoQ) 0.2 mEq/L. Serum creatinine concentration was determined by molecular absorption spectrometry. Detection limit (LoD) was 0.10 mg/dL, and the limit of quantification (LoQ) was 0.15 mg/dL Linear mixed models were used to analyze lithium and creatinine serum concentrations.Time points extractions and lithium dose were included as fixed effects, while the individual mother was included as a random effect to account for repeated measurements.

**Results:**

In total, 1260 lithium and 1174 creatinine serum concentration measurements from 109 pregnancies of 95 women were available. Dose-ajusted serum lithium concentrations (C/D ratio) decreased and average of 23.9% and 27.6% during the first and second trimesters respectively, increased slightly in the third trimester but remained 16.9 % below the prconception time (reference period) and increased postpartum 11% . Serum creatinine concentration evidenced a similar longitudinal pattern during pregnancy (decreased 20%, 26.1% and 22.3% in first, second and third trimester respectively) and also decreased postpartum 4.3%.

**Conclusions:**

We recommend monitoring lithium and creatinine serum concentration once every 4 weeks until 34 weeks of pregnancy, then weekly until delivery, followed in postpartum at day 2±1, (bi-)weekly during the first month, monthly until 6 months postpartum and then every 3-4 months to ensure adequate lithium dosing.

**Disclosure of Interest:**

None Declared

